# Supplementary material for: Modifying Deutsch’s scale illusion for application in music
Source: PLoS One. 2023 Feb 1;18(2):e0280452. doi: 10.1371/journal.pone.0280452 (PMC9891530; doi:10.1371/journal.pone.0280452)
Supplement: Attachment — Submitted filename: Response_to_Reviewers.docx [file pone.0280452.s008.docx]

Response to Reviewer #1:

We appreciate your valuable comments. We hope the revised version makes it clearer to understand.

As added in the manuscript, "local pitch proximity" means "note-to-note pitch proximity," and "laterality" means "differences in ear of input." I have changed the term, "global pitch range" to "overall pitch range" because it is less confusing. To avoid confusion, I have also changed the expression, "other notes" to "other note patterns," and "tonal sequences" to "tone patterns."

Following your advice, I have repeated the Materials and Methods sections along with Results and Discussion for each experiment. Detailed description was added in the Statistical analysis section. In the Materials and Methods section, it is described "The tone patterns that switched between the right and left ears were tested in the same way."

In the statistical analyses, statistical significance was set at p < 0.05, and I have deleted the description of p < 0.01. In Experiment 3, McNemar's chi-squared test was applied.

I have changed the description of the tasks for Experiment 4.

In the General Discussion, I have removed the discussion about objective vs. subjective because it is less important. In the Introduction and the General Discussion, I have added the description of why the Deutsch illusion with music is desirable or interesting.

I especially appreciate your comment concerning the color of the figures because honestly, I had never thought about figures that are friendly to the color blind. For more telling apart, I have changed the green solid line to a green dotted line. I have also changed the description in the figure legend, from "red line" to "red circles and solid line," and "blue line" to "blue Xs and dotted line." The missing figure legend about the green line was also added.
