# Supplementary material for: Modifying Deutsch’s scale illusion for application in music
Source: PLoS One. 2023 Feb 1;18(2):e0280452. doi: 10.1371/journal.pone.0280452 (PMC9891530; doi:10.1371/journal.pone.0280452)
Supplement: Attachment — Submitted filename: Response_to_Reviewers.docx [file pone.0280452.s009.docx]

**Response to Reviewer Comments:**

Thank you for your valuable comments. We sincerely appreciate the time and effort that you have dedicated to providing your valuable feedback on the manuscript. We have been able to incorporate changes to reflect most of the suggestions provided, and hope that the revised version is clearer to understand.

**1. From reading the abstract and the introduction, I thought that there are 2 experiments only. It might be clearer to replace 'in the first half of the experiment' with experiment 1 and 2 and replace 'in the latter half of the study' with experiment 3 and 4. Also, I suggest providing a brief overview of the 4 experiments in introduction.**

**Response:** We replaced the terms “Experiment 1, 2, 3, and 4” with “Experiment 1a, 1b, 2a, and 2b.” We have also used the expression "in the first half of the study" and "in the latter half of the study" in the revised manuscript. A brief overview of the 4 experiments was also provided in introduction.

**2. Line 163 and line 334-335: if p-value was set at 0.05, then the post hoc test did not use Bonferroni-corrected p value? It would be great if test statistics and exact p-values for significant results are reported in results session.**

**Response:** Based on the reviewer’s comments, we realized that the description regarding p values and Bonferroni corrections may have been confusing. We have clarified that the post hoc test did use Bonferroni-corrected p values, and that p values < 0.05 were considered to be statistically significant. The exact p values for significant results have now been reported in the Results sections.

**3. Line 319-320: what is the time period between experiment 3 and 4? How soon did the participants from experiment 3 completed the next experiment?**

**Response:** The time period between Experiment 2a (previously Experiment 3) and 2b (previously Experiment 4) has been specified. Experiment 2b was conducted at least one day after Experiment 2a.
