# Supplementary material for: Modifying Deutsch’s scale illusion for application in music
Source: PLoS One. 2023 Feb 1;18(2):e0280452. doi: 10.1371/journal.pone.0280452 (PMC9891530; doi:10.1371/journal.pone.0280452)
Supplement: Attachment — Submitted filename: Response_to_Reviewers.docx [file pone.0280452.s010.docx]

Response to Reviewer #1:

We greatly appreciate your valuable comments. Following your advice, we have revised the *p*-values.

Response to Reviewer #2:

We greatly appreciate your valuable comments. We understand that the general discussion section was confusing. Following your advice, we have re-written the section. We hope that the revised version of the manuscript has properly addressed your concerns.
